# Management of major depression in outpatients attending a cancer centre: a preliminary evaluation of a multicomponent cancer nurse-delivered intervention

**DOI:** 10.1038/sj.bjc.6601546

**Published:** 2004-01-20

**Authors:** M Sharpe, V Strong, K Allen, R Rush, P Maguire, A House, A Ramirez, A Cull

**Affiliations:** 1 Division of Psychiatry, School of Molecular and Clinical Medicine, University of Edinburgh, Kennedy Tower, Royal Edinburgh Hospital, Morningside Park, Edinburgh EH10 5HF, UK; 2 Cancer Research UK, Edinburgh Oncology Unit, Western General Hospital, Crewe Road, Edinburgh EH4 2XR, UK; 3 Cancer Research UK, Psychological Medicine Group, Christie Hospital NHS Trust, Stanley House, Wimslow Road, Withington, Manchester M20 4BX, UK; 4 School of Medicine, University of Leeds, 15 Hyde Terrace, Leeds LS2 9LT, UK; 5 Cancer Research UK, London Psychosocial Group, Department of Liaison Psychiatry, Adamson Centre for Mental Health, South Wing, St Thomas' Hospital, London SE1 7EH, UK

**Keywords:** major depressive disorder, treatment, nurse, problem-solving therapy

## Abstract

A novel nurse-delivered multicomponent intervention for major depressive disorder (MDD) in cancer outpatients was compared with usual care alone in a nonrandomised matched group design (*n*=30 per group). At the final 6-month outcome, 38.5% (95% CI, 5.4–57%) fewer patients in the intervention group still met the criteria for MDD.

Major depressive disorder (MDD) (American Psychiatric Association, 1994) is associated with an increased symptom burden, greater disability, reduced quality of life and poorer medical outcome (Katon, 1996), and occurs in a substantial proportion of cancer patients (Lynch, 1995; McDaniel *et al*, 1995). There is some evidence for the efficacy of antidepressant drugs (McDaniel *et al*, 1995) and for psychological therapies (Sheard and Maguire, 1999). However, many patients do not receive any potentially effective treatment (Lynch, 1995). We have therefore developed and piloted a cancer nurse-delivered intervention. The aim of this study was to perform a preliminary evaluation of its feasibility and efficacy in oncology outpatients.

## MATERIALS AND METHODS

### Patients and recruitment

We recruited patients with MDD from consecutive attenders at breast, gynaecological, bladder, prostate, testicular and colorectal clinics at the Edinburgh Cancer Centre between September 1999 and September 2000 using a screening procedure described in the companion paper (Sharpe *et al*, 2003).

Patients were excluded if: (a) the oncologist predicted that they would not survive to follow-up or if they had (b) a complicating and uncontrolled medical problem, (c) a complicating major psychiatric diagnosis, (d) a history of continuous depression for more than 1 year prior to cancer diagnosis, (e) difficulty in communicating and (f) were currently receiving active specialist treatment from a psychologist or psychiatrist. When patients were excluded, their doctors were told of their diagnosis of MDD.

### Design and power

The study was a nonrandomised comparison of the outcome of two sequentially recruited cohorts. To detect a difference in the proportions of patients still meeting the criteria for MDD of the magnitude size previously reported in a similar study in primary-care patients (35%) (Katon *et al*, 1996) using a paired analysis, approximately 56 cases (28 in each of the treatment and usual care groups) are required (80% power and significance level of 0.05).

## MEASURES

### Baseline measures

*Demographic and cancer data*: These were collected from medical records.

#### Structured Clinical Interview for DSM-IV (SCID)

The presence of MDD and depressive symptoms was determined during telephone interview using the SCID (First *et al*, 1999). All symptoms counted toward the diagnosis and no judgements were made about their aetiology. Telephone SCID has good agreement with a face-to-face interview (Cacciola *et al*, 1999), and is acceptable to patients (Allen *et al*, 2003).

*Hospital Anxiety and Depression Scale (HADS)* (Zigmond and Snaith, 1983): Anxiety and depressive symptoms were assessed with this 14-item self-rated scale designed for use in the medically ill (see companion paper Sharpe *et al*, 2003).

*Manchester Concerns Checklist* (Harrison *et al*, 1994): Patient's concerns were rated using this 14-item checklist, each concern being rated on a five-point scale from ‘not a worry’ to ‘extremely worried’.

#### Outcome measures

The primary outcome measure was the presence of MDD according to DSM-IV criteria as assessed by the SCID interview and determined from audiotapes and/or notes of the interviews by a consultant psychiatrist (MS) blind to the patient's treatment status. The number of MDD symptoms on the SCID and self-rated scales listed above were secondary outcome measures.

#### Definition of adequate dose of antidepressant drugs

We defined the adequacy of the dose of antidepressant drugs as that specified in the British National Formulary (www.bnf.org), but accepting 75 mg as effective for tricyclic antidepressants (Furukawa *et al*, 2002).

### Treatment conditions

#### Usual care alone

We told the GP, oncologist and the patient about the diagnosis of MDD and the GP was asked to ‘manage the patient as they normally would’.

#### Usual care plus the experimental intervention

These patients also received the nurse-delivered intervention, which comprised:

1. Education about depression (e.g. that the symptoms of depression were a separate problem from the cancer and of the probable effectiveness of treatment).
2. Up to ten 30-min sessions of problem-solving therapy intended to help patients to take a positive and systematic approach to tackling their problems (Wood and Mynors-Wallis, 1997).
3. Patients were encouraged, within the context of problem solving, to consider seeing their GP to discuss antidepressant therapy. Although prescribing was left to the GP, the nurse ensured that if taken, a therapeutic dose of antidepressant was attained and adhered to.
4. Coordinating and monitoring the patient's treatment with respect to the MDD.

The cancer nurse received 6 months training in the intervention and was rated as competent on the basis of videotape recordings of treatment. She received weekly supervision from a consultant psychiatrist during the trial. Treatment sessions took place weekly in the hospital or where necessary at home or by telephone. After treatment, patients were told that they could contact the nurse for ‘booster’ sessions.

### Procedure

All participating patients gave written consent.

#### Allocation to treatment

We assigned patients recruited between 1999 and February 2000 to usual care only and those recruited from March 2000 to August 2000 to the additional intervention.

#### Follow-up

Outcome assessments were at 3 and 6 months by telephone interview and postal self-report questionnaires. At the end of the study period, GPs were notified if their patient still had MDD.

### Statistical analysis

Matching of the groups was by individual baseline variables in the following order: sex, age (10-year bands), presence of active disease and cancer site. The statistical analysis was appropriate for matched pairs. The McNemar test was used to compare proportions and paired *t*-tests to compare means. All tests were two-tailed.

### Ethical approval

Lothian Research Ethics Committee approved the study.

## RESULTS

### Patients and recruitment

We identified 196 patients with MDD by screening. Seven refused further assessment and 39 were ineligible (20 because of chronic depression and 19 for other reasons). Nonparticipation among those approached was 31of 83 (37%) for usual care and 34 of 64 (53%) for the additional intervention. The reasons given were time, distance and a preference not to discuss emotional matters. The participating clinicians were positive about the intervention.

### Matching of groups

After matching 30 usual care only patients to the 30 receiving the intervention, there were no substantial or statistically significant differences between groups on demographic, cancer or psychological variables (see Tables 1

**Table 1:** Demographic and characteristics of depression of the matched groups at baseline

	**Number (percentage) unless otherwise specified**	
	**Intervention (*n*=30)**	**Usual care (*n*=30)**
*Demographics*		
Mean age in years (standard deviation)	58 (10.6)	56 (10.5)
Female	28 (93.3)	28 (93.3)
*Depression*		
Median duration (months) of episode (range)	7 (1–120)	7 (1–96)
*Depression onset in relation to cancer*		
With diagnosis	7 (23.3)	10 (33.3)
With recurrence	3 (10)	2 (6.7)
After diagnosis/recurrence	20 (66.7)	18 (60)
*Self reported previous depression*		
No previous episodes	15 (50)	17 (56.7)
Prescribed antidepressant agent for this episode	15 (50)	5 (16.7)
Currently taking a therapeutic dose of an antidepressant agent^a^	5 (16.7)	3 (10)
Previously received psychological therapy^b^	5 (16.7)	1 (3.3)
Previously used other support services^c^	3 (10)	3 (10)
		
*Cancer*		
Median time (years) since diagnosis (range)	4 (0–19)	3 (0–29)
Site		
Breast^d^	26 (87)	27 (90)
Disease state		
No active disease	24 (80)	24 (80)
Local disease	2 (7)	2 (7)
Metastases	4 (13)	4 (13)
Treatment stage		
Active treatment^e^	8 (27)	2 (7)

a Defined in methods.

b Consulted a psychiatrist/psychologist.

c Other supportive services – breast care nurse, occupational health-care nurse, counsellor, faith-healer and non-NHS charitably funded cancer support centre.

d ther sites were the bladder, prostate, colorectal, thyroid and ovary.

e Long-term hormonal-based therapies such as tamoxifen not included as active treatment.

and 2

**Table 2:** Primary and secondary outcome variables at baseline 3 and 6 months

**Time**	**No. pairs**	**Intervention**	**Usual care only**	**Mean difference (95% CI)**	***t***	**Sig. (*P*-value)**
*Interview (SCID)*						
No MDD^*^						
Baseline	30	0	0			
3 months	28	71 (20)	32 (9)	39.3 (7.9–56)		0.01
6 months	26	81 (22)	42 (11)	38.5 (5.4–57)		0.02
						
Number of MDD symptoms						
Baseline	30	6.4 (1.2)	6.5 (1.3)	0 (−0.7–0.5)	0.3	0.74
3 months	28	3.1 (2.4)	5.5 (2.2)	2.3 (−3.6–−1.1)	3.8	0.001
6 months	26	2.6 (2.3)	4.9 (2.2)	2.2 (−3.8––0.7)	3.0	0.006
						
*Self-Rated Scales*						
HADS anxiety						
Baseline	30	12.9 (3.1)	12.8 (3.6)	0 (−1.9–2.0)	0.04	0.97
3 months	27	7.7 (4.1)	12.6 (3.6)	4.8 (−7.1–−2.6)	4.3	0.000
6 months	26	7.9 (4.7)	11.7 (3.7)	3.8 (−6.6–−0.9)	2.7	0.012
HADS depression						
Baseline	30	10.4 (3.6)	10.3 (4.0)	0 (−1.8–2.0)	0.71	0.94
3 months	27	7.0 (4.4)	10.6 (3.7)	3.5 (−5.9–−1.1)	3.02	0.006
6 months	26	7.0 (4.1)	9.6 (4.7)	2.7 (−5.5–0.1)	1.96	0.061
Number of concerns						
Baseline	30	6.7 (3.5)	6.7 (3.1)	0 (−1.7–1.6)	0.04	0.97
3 months	28	3.9 (4.1)	6.0 (3.7)	2.4 (−4.5–−0.2)	2.3	0.03
6 months	25	3.3 (3.9)	5.7 (4.1)	2.2 (−4.7–0.34)	1.8	0.09

Outcomes expressed as percentages and percentage differences for pairs, and means and mean differences for pairs, with confidence intervals. Statistical significance by paired *t*-tests and McNemar test^*^. ^[2]^All means and (s.d.) except^*^, which are percentages (numbers). CI=confidence intervals; SCID=Structured Clinical Interview for DSM-IV; MDD=major depressive disorder; HADS=Hospital Anxiety and Depression Scale.

). Most had breast cancer and were in a postacute treatment phase. A greater number of patients in the intervention group had received antidepressant drugs prior to recruitment, but only a very small and similar number were taking a therapeutic dose.

### Missing outcome data

Data on the principal outcome was available on 28 pairs at 3 months and 26 pairs at 6 months (four patients died and two were lost to follow-up). There was a small amount of additional missing data on secondary measures due to noncompletion of questionnaires.

### Intervention received

#### Problem-solving therapy

Every patient in the intervention group had an initial assessment, plus between 1 and 13 weekly problem-solving sessions (mean 7.3, s.d. 2.0) of approximately 30 min duration. The duration of treatment ranged from 2 to 16 weeks. Only six patients requested post-treatment ‘booster’ sessions. Only four sessions were conducted at the patients' home and very few by telephone. The treating nurse spent a mean of 6 h with each patient and an additional 4 h on administration, calls to GPs, travel and supervision. The psychiatrist provided 1 h of supervision per week and attended treatment sessions on four occasions. The total time spent per patient treated was therefore approximately 10 h for the nurse and 1 h for the psychiatrist.

#### Antidepressant medication

During the 6-month study period, most (27 of 30; 90%) of the patients in the intervention group were prescribed an antidepressant by their GP, although only 16 (53%) attained a therapeutic dose, largely because of the poor tolerance of side effects. In comparison, only half (16 of 30; 53%) of the patients in the usual care only group were prescribed an antidepressant drug and only (23%) attained a therapeutic dose.

### Other treatments received

One patient in the treatment group and four patients in the usual care only group saw a psychologist or psychiatrist during the study period. A small number of patients in each group saw other counsellors.

### Outcome data

#### Principal outcome

Table 2 shows the percentage of patients who no longer met the criteria for MDD at 3 and 6 months and the number of symptoms of MDD elicited on the SCID interview. There was a substantially and statistically significantly greater improvement in the intervention group at both time points.

#### Secondary outcomes

Table 2 also shows the substantial and statistically significantly greater reductions in the self-rated secondary outcomes, which were greatest at 3 months and largely but not entirely maintained at 6 months.

## DISCUSSION

### Main findings

This preliminary comparison of the nurse-delivered intervention with usual care suggests that it is feasible to train a nurse, to deliver the intervention (although with a high patient refusal rate – see below) and to gain the cooperation of clinicians. It also produces a substantially better outcome for patients. The beneficial effect is apparent at the end of the active treatment phase (3 months) and is largely maintained at the follow-up point (6 months).

### Limitations

Nonrandomised comparisons can overestimate the differences between treatments (Altman and Bland, 1999). There was a relatively low participation rate, although surprisingly, this was not much higher in those offered usual care only than in those asked to participate in the much more time-consuming treatment intervention. The main reason for this is probably that patients were recruited by screening rather than referral. An intervention study for depression after myocardial infarction reported similar findings (Frasure-Smith *et al*, 1997). Participants were predominantly females with inactive breast cancer, limiting the generalisability of the findings. The sample was small leading to wide confidence intervals around the results. Finally, as a single nurse administered the intervention, we cannot conclude that cancer nurses in general could necessarily be trained to deliver the treatment effectively.

### Other studies

Oncologists miss many cases of depression (Fallowfield *et al*, 2001). However, simply feeding back the findings of screening does not seem to help (McLachlan *et al*, 2001). We therefore need to not only improve the identification of depression but also the provision of treatment. Specialist psychiatrists and psychologists are in short supply. One potential solution that requires further evaluation is for the medical and nursing oncology staff to play a greater role (Stiefel *et al*, 2001). It is therefore surprising that there have been few trials of cancer nurse delivered interventions for depression. Early studies found that simple counselling by specialist nurses did not prevent depression, but that better recognition and subsequent treatment by a psychiatrist improved outcome (Maguire *et al*, 1980; Maguire *et al*, 1985). We are not aware of any previous studies of the management of established MDD comorbid with cancer by oncology nurses or other oncology clinic staff.

### Implications

The results of this preliminary study indicate that depression management systems that combine screening and intervention are worthy of further development and evaluation in randomised trials.

## FUNDING

The NHS Research and Development/Cancer Research Campaign (now Cancer Research UK) Cancer Research Programme funded this study.
